# Inflammatory Biomarkers Demonstrate Predictive Capacity for Mortality in COVID-19-Related ARDS Patients Receiving High-Dose Corticosteroids: A Longitudinal Analysis

**DOI:** 10.2147/JIR.S502188

**Published:** 2025-02-18

**Authors:** Katrijn Daenen, Anders Boyd, Jilske A Huijben, Sara C M Stoof, Lieuwe D J Bos, Diederik Gommers, Eric C M van Gorp, Virgil A S H Dalm, Henrik Endeman

**Affiliations:** 1 Department of Intensive Care, Erasmus University Medical Center, Rotterdam, The Netherlands; 2 Department of Viroscience, Erasmus University Medical Center, Rotterdam, The Netherlands; 3 Department of Infectious Diseases, Public Health Service of Amsterdam, Amsterdam, The Netherlands; 4 Stichting HIV Monitoring, Amsterdam, The Netherlands; 5 Infectious Diseases, Amsterdam University Medical Centers, Location University of Amsterdam, Amsterdam, The Netherlands; 6 Department of Intensive Care, Amsterdam University Medical Centers, Location Academic Medical Centre, University of Amsterdam, Amsterdam, The Netherlands; 7 Laboratory of Experimental Intensive Care and Anesthesiology, Amsterdam University Medical Centers, Location Academic Medical Centre, University of Amsterdam, Amsterdam, The Netherlands; 8 Department of Internal Medicine, Erasmus University Medical Center, Rotterdam, The Netherlands; 9 Department of Immunology, Erasmus University Medical Center Rotterdam, Rotterdam, The Netherlands; 10 Department of Internal Medicine, Division of Allergy & Clinical Immunology, Erasmus University Medical Center, Rotterdam, The Netherlands; 11 Department of Intensive Care, OLVG, Amsterdam, The Netherlands

**Keywords:** infectious diseases, virology, severity, prediction

## Abstract

**Purpose:**

Patients with coronavirus disease 2019 (COVID-19)-related acute respiratory distress syndrome (ARDS) who lack clinical improvement are frequently treated with high-dose corticosteroids (HDS). Since HDS is used to reduce hyperinflammation in these patients, levels of (pro-)inflammatory biomarkers after commencing HDS treatment could be useful in predicting mortality. This study aims to evaluate biomarker levels after commencing HDS over time, along with their capacity to predict mortality.

**Patients and Methods:**

This retrospective cohort study included patients with COVID-19 ARDS treated with HDS in the intensive care unit (ICU) at an academic hospital in the Netherlands between March 2020-March 2022. Inflammatory biomarkers (ie, C-reactive protein (CRP), D-dimer, ferritin, leukocyte count, interleukin-6 (IL-6), lactate dehydrogenase (LDH), neutrophil-to-lymphocyte ratio (NLR), and procalcitonin (PCT)) were assessed daily from start of HDS (ie baseline) until day 7. Associations between biomarker levels and all-cause-hospital-mortality were evaluated each day using logistic regression, with cut-offs identified by optimizing sensitivity (Se) and specificity (Sp).

**Results:**

Of the 122 patients included, 53 (43.4%) died during hospitalization. HDS was initiated for a median 7 days (IQR=1–11) after ICU admission. At baseline, a moderately high predictive capacity for mortality was observed at a ferritin level >1281 µg/L (Se=62%/Sp=64%), leukocyte count >13.7 × 109/L (Se=42%/Sp=79%), and NLR >12.1 (Se=61%/Sp=77%). During follow-up, CRP >50 mg/L on day 6 (Se=50%/Sp=75%) and >42 mg/L on day 7 (Se=50%/Sp=75%), ferritin >1082 µg/L on day 6 (Se 63%/Sp=71%) and >1852 µg/L on day 7 (Se=31%/Sp=79%), IL-6 >67 mg/L on day 7 (Se=56%/Sp=79%) and LDH >396U/L on day 6 (Se=38%/Sp=83%) and >373 U/L on day 7 (Se=47%/Sp=72%) showed moderate capacity to predict mortality. NLR was consistently associated with mortality for all days, except day 1 (Se=36–68%/Sp=72-92%).

**Conclusion:**

In COVID-19 ARDS patients receiving HDS, several clinically available inflammatory biomarkers moderately predicted all-cause-hospital-mortality after the start of HDS, particularly on days 6 and 7. NLR demonstrated the most consistent association with mortality over time. The use of these markers requires validation in larger cohorts.

## Introduction

Acute Respiratory Distress Syndrome (ARDS) is a life-threatening condition, with mortality rates that persist around 40% despite significant biomedical efforts.^1–3^ In late 2019, a novel coronavirus, severe acute respiratory syndrome coronavirus 2 (SARS-CoV-2), emerged, which caused Coronavirus disease 2019 (COVID-19). By 2020, SARS-CoV-2 became a pandemic and resulted in significantly increasing numbers of patients with ARDS requiring ICU admission.^4^ This population of ARDS patients offered a unique opportunity to evaluate treatments and clinical prediction strategies on a larger scale. ARDS is characterized by the acute onset of inflammatory hypoxemic respiratory failure, where activation of the immune system and the consequent release of (pro-)inflammatory cytokines play an important role.^5^,^6^ Research before the COVID-19 pandemic has shown that higher plasma levels of inflammatory biomarkers in ARDS are associated with a higher risk of mortality.^7^,^8^ Studies describing the immunological features of patients with COVID-19 ARDS have reported elevated levels of peripheral blood inflammatory markers, including procalcitonin (PCT), C-reactive protein (CRP), interleukin-6 (IL-6), and lactate dehydrogenase (LDH), as well as increased neutrophil-to-lymphocyte ratios (NLR).^9–11^ Similar to non-COVID-19 ARDS, the excessive release of these cytokines during COVID-19 ARDS is also strongly associated with the risk of multiple organ failure and mortality.^12^ The inflammatory environment in the lungs causes endothelial damage and triggers the release of activated tissue factor from endothelial cells, macrophages, and neutrophils, thereby enhancing the activation of the coagulation cascade in the pulmonary microvasculature. This is reflected by elevated levels of markers of endothelial damage and coagulation, such as D-dimer and ferritin, which are frequently increased in COVID-19 ARDS and are associated with poorer outcomes.^13^,^14^

For decades, corticosteroids have been used in the treatment of patients with non-COVID-19 ARDS because of their strong anti-inflammatory effects, with current ARDS guidelines recommending 1 mg per kg bodyweight methylprednisolone per day.^15–17^ Corticosteroids have also become the first-line immunosuppressive therapeutic agents for patients with COVID-19, and have clearly demonstrated reduced mortality in those receiving oxygen therapy or invasive mechanical ventilation.^18^,^19^ Various comparative trials have studied the optimal corticosteroid dosage for treatment in COVID-19^20–23^ with current guidelines recommending 6 mg of dexamethasone per day for 10 days when oxygen therapy is required, regardless of the level of inflammation or disease severity.^18^

When patients experience progressive clinical deterioration despite standard corticosteroid treatment, clinicians often resort to HDS following ARDS guidelines in an attempt to mitigate severe hyper-inflammation. Successful therapeutic responses to HDS would likely involve a reduction in inflammation and consequently lower the risk of mortality. As such, inflammatory markers could potentially serve as indicators of clinical outcomes not only at baseline before administration but also in the days following the start of HDS treatment.

This study aims to assess the role of inflammatory markers after commencing HDS treatment in patients with COVID-19 ARDS. To this end, we first quantified the changes in levels of inflammatory markers over time. We then evaluated the association between inflammatory marker levels and all-cause mortality each day. Finally, we determined the capacity of inflammatory markers at baseline and during follow-up to predict mortality after commencing HDS treatment.

## Material and Methods

### Study Design

We conducted a retrospective, single-center, longitudinal cohort study. We analyzed data from patients with COVID-19 ARDS who were admitted to the Erasmus University Medical Center (Rotterdam, the Netherlands) between 28 March 2020 and 22 February 2022. Patients were included if they were at least 18 years of age, were admitted to the ICU, had either a Polymerase Chain Reaction (PCR)-confirmed SARS-CoV-2 infection or high clinical suspicion of COVID-19, and received HDS at some point during ICU admission. Patients who died or were discharged within 2 days after admission were excluded from the study.

HDS was defined based on specific dosing thresholds for each corticosteroid used, if prescribed for the indication of COVID-19 (Supplementary Table 1). At the beginning of the pandemic, the decision to start HDS was made by the physician following multidisciplinary discussion. From April 7, 2021, this decision was based on protocols established at the Erasmus University Medical Center, which were largely based on existing ARDS guidelines.^24^ The protocol recommended HDS for patients with moderate COVID-19 (stage IIb), characterized by pulmonary involvement with hypoxemia, or severe COVID-19 (stage III), characterized by extrapulmonary systemic hyperinflammation. If patients were diagnosed with moderate or severe COVID-19 within 14 days of symptom onset, the treatment regimen consisted of 1 to 3 days of 1000 mg methylprednisolone. The distinction between stages was based on respiratory parameters, imaging findings, and systemic inflammation assessed through inflammatory markers. Patients met the criteria for stage IIb or III if they had a PaO_2_/FiO_2_ ratio <300 mmHg or compliance <60 mL/cmH_2_O.^25^

The study was approved by the local Medical Ethics Review Committee under protocol number MEC-2017-417 and conducted according to the principles of the Declaration of Helsinki. Due to the urgency of conducting research in patients with COVID-19, an exemption for consent was approved by the Medical Research Ethics Committee at the Erasmus University Medical Center. An opt-out informed consent procedure (MEC-2022-0297) was used and patients who expressed objections to participation were excluded from the study.

### Data Collection

Patient data, including demographics, body mass index (BMI), vital signs, comorbidities, APACHE-IV, SOFA-score, and laboratory tests, were extracted from the electronic health record upon ICU admission and were collected during follow-up. The PaO_2_/FiO_2_ ratios were calculated using the PaO_2_ and FiO_2_ measurements collected closest to 8 AM on the same day.

We selected markers of inflammation, coagulation, and endothelial injury, each of which has been demonstrated in previous research to correlate with disease severity and mortality in patients with COVID-19.^9^,^10^ These biomarkers included the following: C-reactive protein (CRP), D-dimer, ferritin, leukocyte count, interleukin-6 (IL-6), lactate dehydrogenase (LDH), neutrophil-to-lymphocyte ratio (NLR), and procalcitonin (PCT). Values closest to 8 AM on each day of follow-up were used in the analysis. Markers were measured using standard laboratory assays during the follow-up period and were collected at the discretion of the ICU medical staff as part of routine care. Blood samples were obtained at the ICU and centrifuged (3000 N Relative centrifugal force (Rcf)) at room temperature for 5 minutes. The serum was analyzed on the routine analyzer of the clinical chemistry laboratory of the Erasmus University Medical Center (Roche Cobas 8000 system, Roche Diagnostics, Rotterdam, the Netherlands). CRP and D-dimer levels were measured using a turbidimetric method (C502 Cobas® assay^26^) and ferritin, PCT, and IL-6 were measured using Electro-Chemi Luminescent Immuno Assay (ECLIA) tests (E801 Cobas® assay).^27^ Leukocyte counts, neutrophil counts, and lymphocyte counts were determined using Sysmex automated cell counters, and May-Grünwald/Giemsa staining and microscopic examination were employed for visual confirmation. LDH levels were quantified through enzymatic analysis.

### Study Outcome

All-cause mortality was the primary outcome of the analysis. Patients were monitored from the time of admission to the ICU until hospital discharge. Follow-up monitoring of the study outcome did not occur after hospital discharge, and thus all deaths were in-hospital.

### Statistical Analysis

Individual follow-up began at the date of commencing HDS treatment (ie, baseline) and continued until either hospital discharge or death, whichever occurred first. Variables at baseline were summarized and compared between survivors and non-survivors using a Kruskal–Wallis test for continuous variables and Pearson’s *χ*^2^ test for categorical variables.

To quantify the changes in biomarkers after commencing HDS treatment, levels were summarized using medians and interquartile ranges (IQR) at baseline and each day during follow-up until day 7. Changes in biomarker levels over time were tested using a Kruskal–Wallis test. These analyses were stratified among survivors and non-survivors.

To assess the association between inflammatory biomarker levels and mortality, the probability of death in the ICU was modeled using separate logistic regression models for each continuous biomarker as a covariate. The odds ratios (OR) and 95% confidence interval (CI) of the odds of mortality per unit increase in biomarker were then estimated from this model. This model was run on data at baseline and each day during follow-up until day 7. Patients with missing data on the day analyzed were removed from the model.

Using the logistic regression models above, the sensitivity (Se), specificity (Sp), and % correctly classified were calculated at each level of individual biomarkers using the “roctab” command in STATA. The level of biomarker that resulted in the highest % of correctly classified patients was selected as the biomarker cut-off. Given that we preferred to decrease the proportion of false-positive results rather than false-negative ones, we selected cut-offs bearing the same % of correctly classified patients with the highest specificity. Using these biomarker cut-offs, we calculated the Se, Sp, positive predictive value (PPV), negative predictive value (NPV), positive likelihood ratio, and negative likelihood ratio for each biomarker as a classifier for mortality. Again, this analysis was run on data at baseline and each day during follow-up until day 7, and patients with missing data on the day analyzed were removed from the model.

All analyses were performed in STATA (v18.0, College Station, TX). A p-value of <0.05 was considered statistically significant. Due to the exploratory nature of these analyses, p-values were not corrected for multiple comparisons.^28^

## Results

### Description of the Study Population

**Figure 1:**
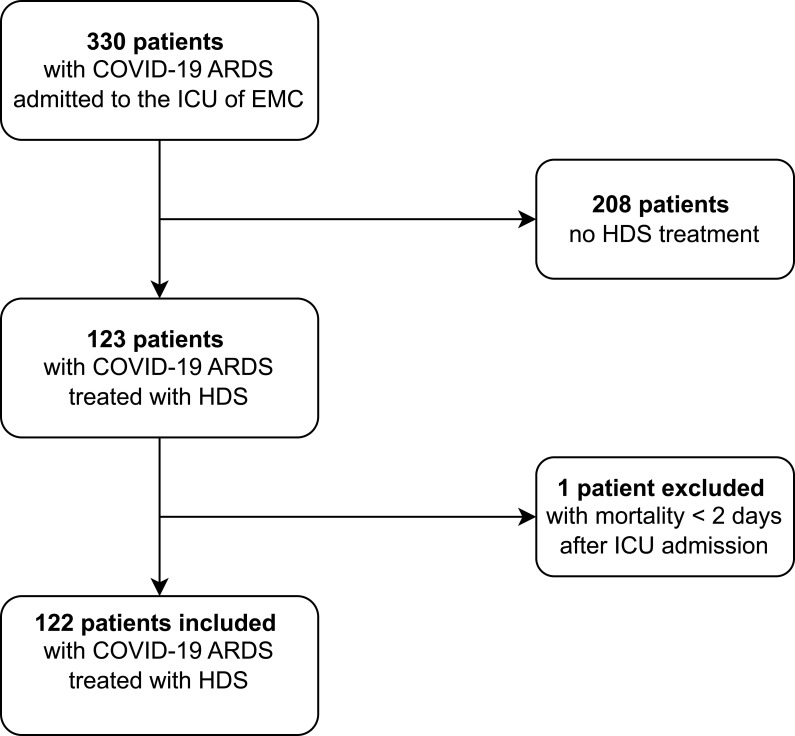
Flow chart of patient selection. Flow chart of selecting patients included in analysis. All patients had COVID-19 ARDS and were admitted to the intensive care unit (ICU) of the Erasmus University Medical Center (EMC) during the study period (March 28, 2020 to February 22, 2022).

Between 28 March 2020 and 22 February 2022, a total of 330 patients with COVID-19 ARDS were admitted to the ICU and screened for inclusion in the study. Of these, 123 received HDS and fulfilled eligibility criteria (Figure 1). One patient died within two days after ICU admission and commencing HDS treatment and was thus excluded from our study. In total, 122 patients were included for analysis.

Median age was 65 years (IQR=58–70), median BMI was 28.3 kg/m2 (IQR=25.1–32.4), and 83.6% of the patients were male (Table 1). HDS treatment was either started on the day of ICU admission (n=14, 11.5%) or a median 7 days (IQR=1–11) after ICU admission. One hundred and eighteen patients (96.7%) received the standard 3-day HDS regimen, while 2 (1.6%) and 2 (1.6%) patients were treated with HDS for shorter (ie, <3 days) or longer (ie, >3 days) durations, respectively. Of all included patients, 97% were treated with three days of 1 gram of methylprednisolone.

**Table 1:** Patient Characteristics at Baseline, Stratified on Survival Status During Follow-up

Patient Characteristics	All Patients	Survival Status		*p* Value
		Survivors	Non-Survivors	
	*n*=122	*n*=69	*n*=53	
Age, years	65 [58–70]	62 [57–67]	67 [61–72]	0.002
Male sex at birth	102 (83.6%)	55 (79.7%)	47 (88.7%)	0.22
BMI, kg/m^2^	28.3 [25.1–32.4]	30.1 [25.4–33.1]	27.7 [25.0–30.4]	0.022
Current smoking	4 (9.5%)	3 (10.7%)	1 (7.1%)	0.99
**Comorbidities**				
Any comorbidity	46 (37.7%)	23 (33.3%)	23 (43.4%)	0.27
Peripheral vascular disease	3 (2.5%)	2 (2.9%)	1 (1.9%)	0.99
Chronic heart failure	3 (2.5%)	1 (1.5%)	2 (3.8%)	0.58
Myocardial infarct	10 (8.2%)	5 (7.3%)	5 (9.4%)	0.75
Pulmonary disease	8 (8.6%)	3 (5.5%)	5 (13.2%)	0.27
Neurological disease	3 (3.2%)	3 (5.4%)	0 (0%)	0.27
Renal disease	10 (10.5%)	4 (7.1%)	6 (15.4%)	0.31
Diabetes mellitus	23 (24.2%)	10 (17.9%)	13 (33.3%)	0.09
Immunodeficiency	9 (9.7%)	5 (8.9%)	4 (10.8%)	0.99
Malignancy	11 (11.6%)	5 (8.9%)	6 (15.4%)	0.35
**Disease severity**				
APACHE-IV predicted mortality score at admission	0.229 [0.142–324]	0.226 [0.131–0.324]	0.245 [0.155–0.323]	0.24
SOFA score	7 [5–10]	6 [5–8]	8 [6–12]	0.001
P/F ratio, kPa	19.1 [12.8–25.4]	21.6 [14.8–25.8]	14.2 [11.0–21.7]	0.008
Length of ICU stay, days	23 [14–36]	27 [17–41]	20 [12–32]	0.029

**Notes**: Baseline was defined as the date of start high-dose corticosteroid treatment and mortality was assessed at hospital discharge. All continuous variables are reported as median [IQR] and all categorical variables as counts and percentages. Survivors and non-survivors were compared using a Kruskal–Wallis test for continuous variables and Pearson’s *χ*2 test for categorical variables.

**Abbreviations**: BMI, body mass index; APACHE, acute physiology, age, and chronic health evaluation; P/F, PaO_2_/FiO_2_.

Patients were followed a median 23 days (IQR=14-36), totaling 11.2 person-years of observation (PYO). During follow-up, 53 (43.3%) patients died a median 29 days (IQR=20-43) after hospital admission. The incidence rate of death was 0.39 per person-months of observation (95% CI=0.29–0.50). Patient characteristics at baseline are compared between survivors and non-survivors in Table 1. Non-survivors were significantly older (p=0.002) and had a lower BMI (p=0.022) than survivors. Moreover, SOFA scores were higher (p=0.001) and PaO_2_/FiO_2_ ratios were lower (p=0.008) in non-survivors compared to survivors, indicating greater disease severity in non-survivors at the time of commencing HDS treatment. There were no significant differences in the proportion of patients with various comorbidities.

### Biomarker Levels Over Time After Commencing HDS Treatment

**Figure 2:**
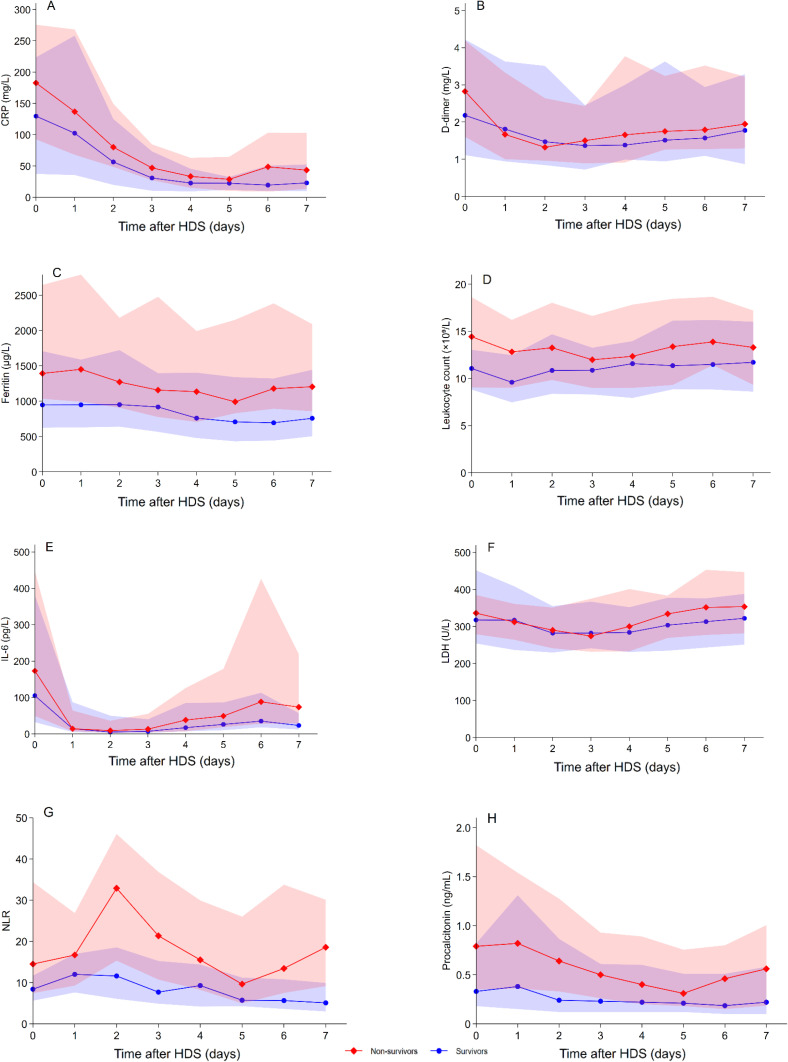
Changes in median biomarker levels over time high-dose corticosteroid treatment. Biomarker levels in peripheral blood over time after commencing high-dose corticosteroid treatment in patients with COVID-19 ARDS are shown for survivors (in blue) and non-survivors (in red). The biomarkers measured include, CRP (**A**), D-dimer (**B**), ferritin (**C**), leukocyte count (**D**), IL-6 (**E**), LDH (**F**), NLR (**G**), and procalcitonin (**H**). Dots represent median levels, and shaded areas indicate interquartile ranges.

**Abbreviations**: CRP, C-reactive protein; IL-6, interleukin-6; HDS, high-dose corticosteroids; LDH, lactate dehydrogenase; NLR, neutrophil-to-lymphocyte ratio.

The median (IQR) values of CRP, D-dimer, ferritin, leukocyte count, IL-6, LDH, NLR, and procalcitonin during follow-up among survivors and non-survivors are shown in Figure 2. Most biomarkers demonstrated an immediate decrease within the first two days after commencing HDS treatment, with the exception of NLR. Significant decreases were observed for both survivors and non-survivors in CRP (*p*=0.0001 and *p*=0.0001, respectively), IL-6 (*p*=0.0001 and *p*=0.0001, respectively), NLR (*p*=0.0001 and *p*=0.028, respectively); while only survivors had significant decreases in ferritin (*p*=0.039) and only non-survivors had significant decreases in LDH (*p*=0.027) and procalcitonin (*p*=0.012).

### Association Between Biomarker Levels and Mortality

**Figure 3:**
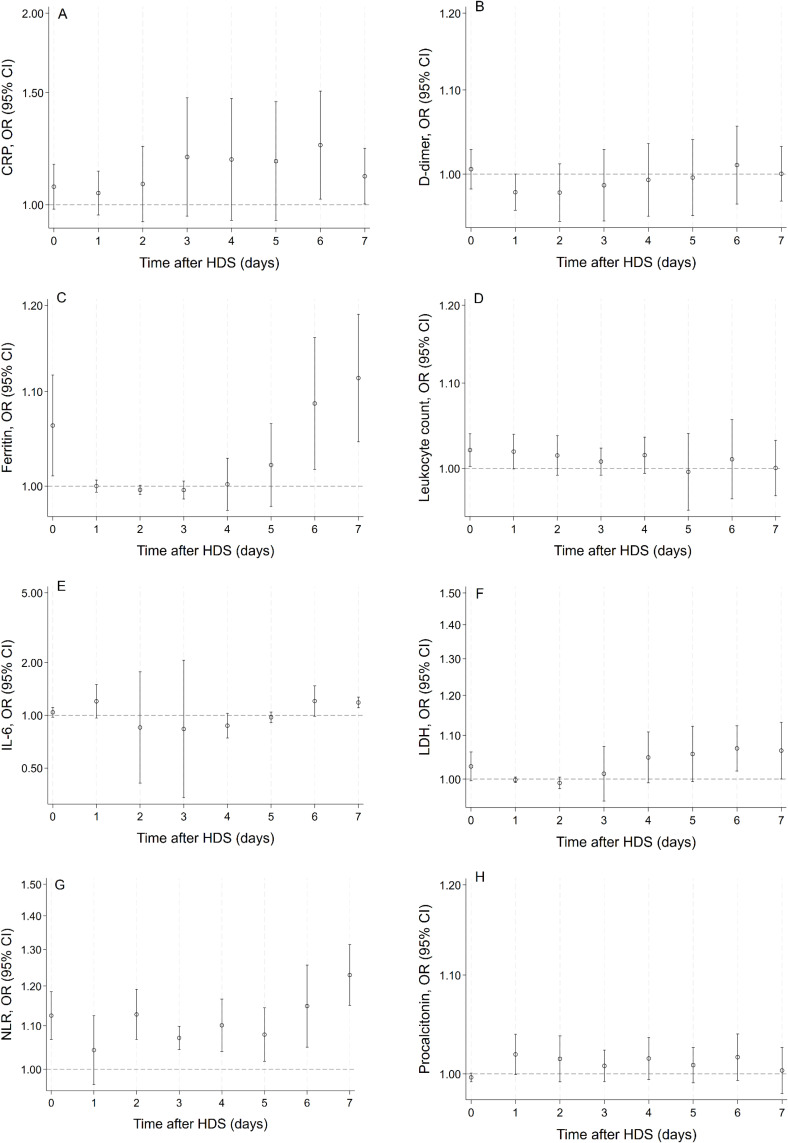
Association of biomarker levels and all-cause mortality per day during high-dose corticosteroid treatment. Odds ratios (in open circles) and their 95% confidence intervals (in bars) for biomarker levels on mortality over time after commencing high-dose corticosteroid treatment in patients with COVID-ARDS are shown for CRP (**A**), D-dimer (**B**), ferritin (**C**), leukocyte count (**D**), IL-6 (**E**), LDH (**F**), NLR (**G**), and procalcitonin (**H**). OR for IL-6 and ferritin was calculated per 1000*pg/mL and µg/L increase, respectively, CRP and LDH per 100*mg/L and U/L increase, respectively, and NLR per 10*units of ratio increase.

**Abbreviations**: CRP, C-reactive protein; IL-6, interleukin-6; HDS, high-dose corticosteroids; LDH, lactate dehydrogenase; NLR, neutrophil-to-lymphocyte ratio; OR, odds ratio.

The OR and 95% CI comparing the odds of death for a one-unit increase in biomarker levels are presented for each of the first seven days following the initiation of HDS treatment in Supplementary Table 2 and are visualized in Figure 3. At baseline, significant associations were observed with ferritin (*p*=0.019), leukocyte count (*p*=0.028), and NLR (*p*<0.0001). Over time, ferritin (on days 6 and 7) and NLR (on all days except for day 1) remained significantly associated, while other markers became significantly associated with mortality: CRP on days six and seven (*p*=0.031 and *p*=0.045, respectively); LDH levels on days 6 and 7 (*p=*0.0080 and *p=*0.045); and IL-6 on day 7 (*p*<0.0001). D-dimer and procalcitonin did not demonstrate significant associations with mortality on any day.

### Predictive Capacity of Biomarker Levels on Mortality

**Figure 4:**
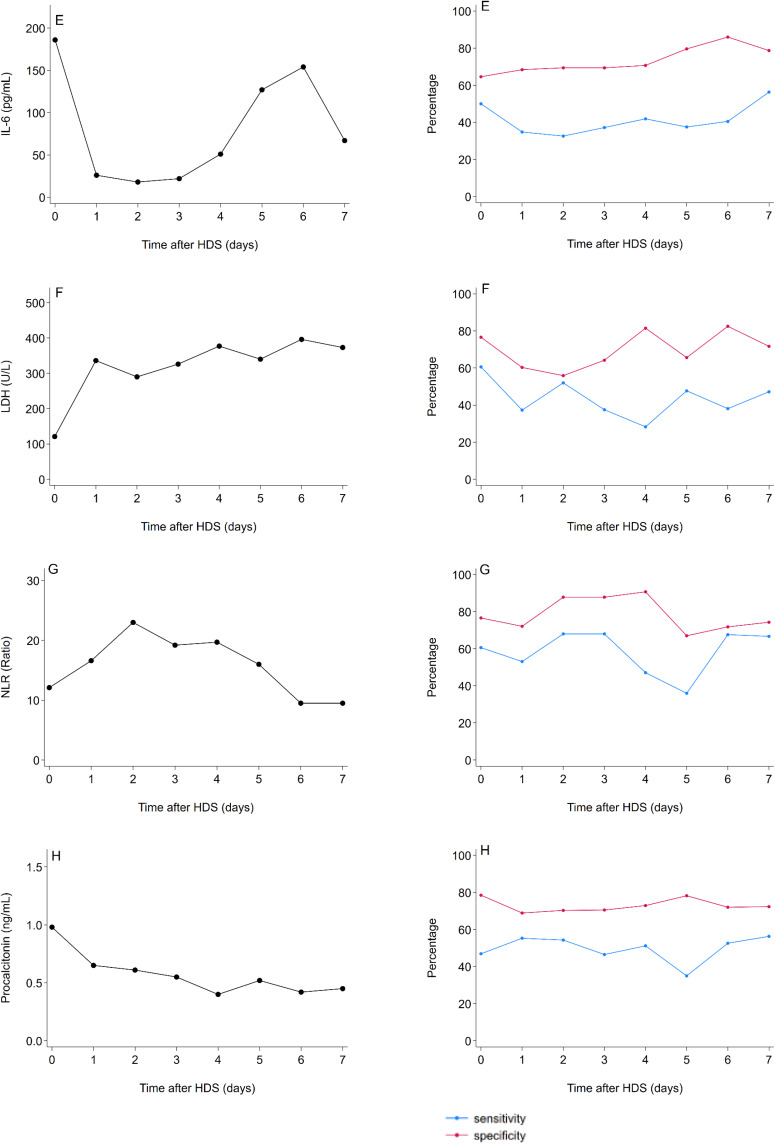
Optimal cut-off biomarker levels in predicting mortality per day during high-dose corticosteroids. Optimal cut-off levels (in black dots) on each day after commencing HDS treatment in predicting mortality are shown in the left column for CRP (**A**), D-dimer (**B**), ferritin (**C**), leukocyte count (**D**), IL-6 (**E**), LDH (**F**), NLR (**G**), and procalcitonin (**H**). Corresponding sensitivity (in blue) and specificity (in red) of markers above the daily optimal cut-off are visualized over time as dots in the right column.

**Abbreviations**: CRP, C-reactive protein; IL-6, interleukin-6; HDS, high-dose corticosteroids; LDH, lactate dehydrogenase; NLR, neutrophil-to-lymphocyte ratio.

Optimal cut-off levels for CRP, D-dimer, ferritin, leukocyte count, IL-6, LDH, NLR, and procalcitonin on each day after commencing HDS treatment in predicting mortality are shown in the left column of Figure 4. Over time, cut-offs to predict mortality for CRP, ferritin, and NLR initially increased, followed by a decrease on day 2 for CRP, and on day 3 for ferritin and NLR. Cut-offs for procalcitonin gradually decreased over the first seven days following commencing HDS treatment, whereas LDH levels remained stable D-dimer, leukocyte count, and IL-6 cut-offs had substantial fluctuations over time.
**Figure 4:**
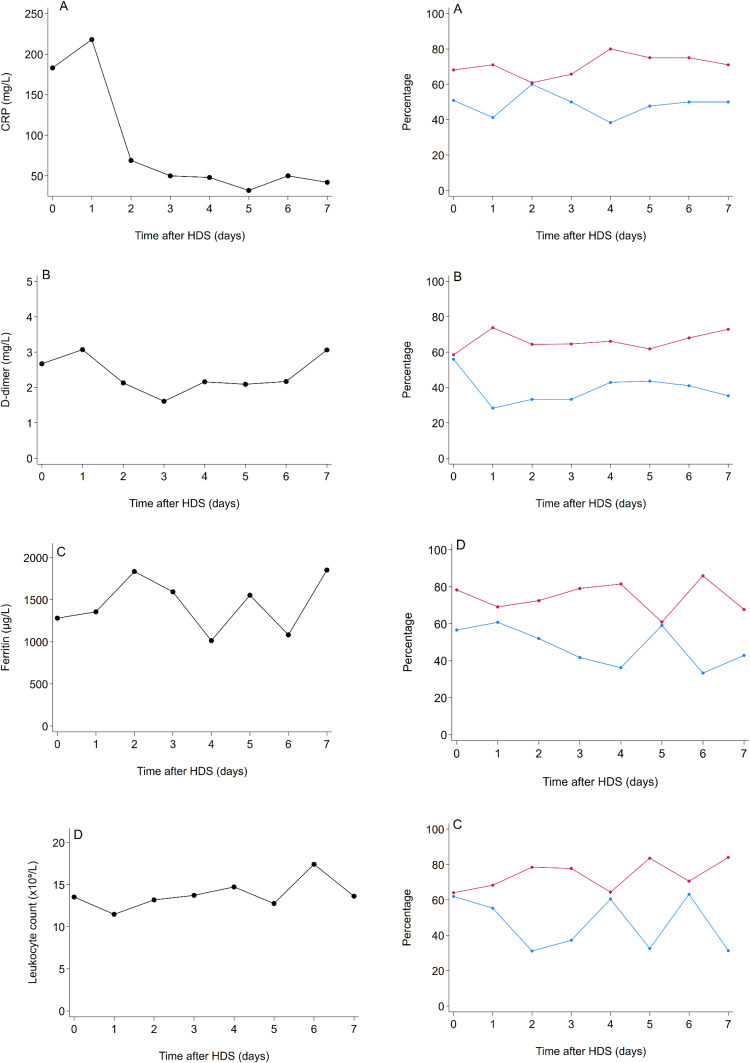
Continued.

The corresponding Se and Sp of markers above the daily optimal cut-offs in predicting mortality are also visualised over time in the right column of Figure 3. When measured at baseline, ferritin >1281 µg/L had the highest sensitivity (Se=62%, 95% CI=47.2%-75.3%) and procalcitonin with >0.98 ng/mL had the highest specificity (Sp=79%, 95% CI=66.5%-87.7%) in predicting mortality during follow-up. There were no consistent changes in Se and Sp of these cutoffs in predicting mortality during follow-up, ranging from 38% (95% CI=25%-54%)-60%(95% CI=45%-74%) and 61%(95% CI=48%-72%)-80%(95% CI=68%-89%) for CRP, 28%(95% CI=16%–44%)–56%(95% CI=41%–70%) and 59%(95% CI=46%–71%)–74%(95% CI=61–84%) for D-dimer, 31%(95% CI=18%–47%)-63%(95% CI=46%–78%) and 64%(95% CI=51%–76%)-84%(95% CI=71%–92%) for ferritin, 33%(95% CI=20%–50%)-61%(95% CI=46%–74%) and 61%(95% CI=48%–73%)–86%(95% CI=75%–93%) for leukocyte count, 33%(95% CI=19%–49%)–56%(95% CI=38%–74%) and 65%(95% CI=52%–76%)–86% (95% CI=73%–94%) for IL-6, 28%(95% CI=16%–48%)–61%(95% CI=51%–73%) and 56%(95% CI=43%–68%)–83%(95% CI=71%–91%) for LDH, 36%(95% CI=18%–58%)–68%(95% CI=49%–83%) and 72%(95% CI=56%–85%)–91%(95% CI=78%–97%) for NLR, and 35%(95% CI=21%–52%)–56%(95% CI=38%–74%), and 69%(95% CI=56%–80%)–79%(95% CI=67%–88%) for procalcitonin, respectively.

Exact values of optimal cut-offs are provided in Supplementary Table 3, along with their corresponding Se, Sp, PPV, NPV, and positive and negative likelihood ratios.

## Discussion

In this longitudinal study, we investigated the use of inflammatory biomarkers as outcome indicators in patients with COVID-19 ARDS receiving HDS treatment. Our analysis revealed significant changes in biomarker levels following the HDS treatment, with an initial decrease in median levels for all biomarkers, except NLR, among both survivors and non-survivors. Interestingly, certain inflammatory biomarkers demonstrated a higher predictive capacity for mortality later in the follow-up period, particularly on days six and seven. NLR consistently showed a strong association with mortality across all time points, except for day 1, exhibiting higher sensitivity and specificity compared to the other biomarkers. This study illustrates that inflammatory biomarkers, and especially NLR, could be of clinical value after commencing HDS treatment when evaluating mortality in this patient population.

Similar to previous studies,^9^,^10^,^12^ we found significant associations between increased levels of NLR, ferritin, and leukocyte count, measured at baseline, and mortality. After commencing HDS treatment, almost all biomarkers demonstrated an initial decrease in median biomarker concentrations during a regimen consisting of three days of 1000 mg methylprednisolone followed by a tapering phase with prednisone. This decrease could be the result of the anti-inflammatory and cytokine-inhibiting effects of corticosteroids.^16^,^29^ The changes in levels of biomarkers may affect their association with mortality. Indeed, in our study, the mortality associations seen at baseline transiently disappeared on day 1, but reappeared on day 2 for NLR, and day 6 for ferritin. Additionally, increased levels of CRP and LDH became significantly associated with mortality on days 6 and 7, and IL-6 on day 7. This observation highlights the added value of using markers not only at baseline but also during the course of follow-up.

Importantly, the optimal cut-offs to predict mortality also fluctuated over time, albeit with comparable Se and Sp. These changes should be considered if clinicians wish to predict mortality at different days after commencing HDS. This longitudinal analysis does, however, have a certain caveat. Patients with missing information on inflammatory markers during follow-up did not have data because they either died or improved sufficiently enough to be discharged from the ICU. This type of censoring means that the target population consists of patients who survived until a given day and are still in ICU, while mortality outcomes reflect those occurring in the time frame after the day of measurement. Notwithstanding the potential for survival bias, the results stratified on days after commencing HDS treatment would be considered generalizable to those specifically remaining in ICU care.

ARDS is characterized by acute respiratory failure presumed to result from a hyperinflammatory state, which is initiated by proinflammatory macrophages and neutrophils. Consequently, the degree of inflammation, as measured by the levels of proinflammatory cytokines in the systemic compartment, would increase the risk of respiratory failure and mortality.^30^ Previous ARDS research has identified two distinct subphenotypes: hyper-inflammatory and hypo-inflammatory, with the former characterized by excessive cytokine release (such as IL-6) and associated with higher mortality rates.^7^,^31^ Notably, corticosteroids have primarily shown survival benefits in COVID-19 patients with ARDS exhibiting this hyperinflammatory subphenotype.^32^ While our findings also show that increased inflammatory markers, such as IL-6 and CRP, are associated with a higher risk of mortality, it remains unclear what is driving this association between hyper-inflammation and mortality. Higher levels of inflammation could indicate a lack of treatment efficacy from the steroid itself or from suboptimal timing or duration of corticosteroid therapy.^25^,^33^ For example, the increase in NLR on day 2 in the non-survivor group and its strong association with mortality might suggest persistent, uncontrolled inflammation despite HDS administration. Alternatively, higher inflammation could be attributed to other causes, such as superinfections.^34–36^ Patients exhibiting increasing NLR, ferritin, CRP, LDH, or IL-6 on days six or seven may require additional diagnostics to identify potential superinfections or consideration of alternative immunomodulatory treatment strategies to optimize care. However, the lack of information on specific causes of death in our study prohibits any inference on these latter causes of inflammation. NLR emerged as the biomarker with the most consistent association with mortality in our cohort of patients with COVID-19 ARDS. Previous research has also consistently linked NLR to poorer prognosis in COVID-19.^37–39^ In response to physiological stress, such as infections, endogenous cortisol is released, leading to an increase in neutrophils and a decrease in lymphocytes, irrespective of corticosteroid treatment.^40^ The excessive cytokine release that occurs in patients with COVID-19 ARDS, as evidenced by the high levels of inflammatory markers (eg, IL-6) at baseline in our cohort, contributes to lymphocyte exhaustion.^41^ Unfortunately, we do not have data on leukocyte subpopulations to further support these hypotheses. Additionally, exogenous corticosteroids promote neutrophil survival, accentuating neutrophil-mediated inflammation,^42^ while HDS induce significant lymphocyte apoptosis.^43^ These processes collectively result in a further elevation of NLR. As NLR shows an association with mortality throughout the entire study period with the highest sensitivity and specificity, we consider it the best candidate biomarker to potentially serve as an indicator of poor outcomes during or shortly after HDS administration.

Our study has several limitations. First, the absence of a standardized protocol for HDS during the early part of the inclusion period means that the target population of those receiving HDS varied over time. Some patients who died early in their ICU stay could have benefited from earlier HDS, potentially resulting in a survival bias. This limitation is, however, shared across other studies from this period. Second, roughly one in four patients also received immunomodulatory therapy in the form of the IL-6R inhibitor tocilizumab, which we could not account for in our analyses due to data limitations. This treatment may have influenced the biomarker kinetics of IL-6 and CRP in these patients. Third, some patients in our study received HDS during at later phases of ARDS, a stage characterized more by fibrosis than by cellular inflammation, during which its effectiveness has been shown to be limited or unbeneficial for outcomes.^33^ Including these patients will likely bias kinetics towards slower rates and cutoffs to predict mortality towards higher levels. Finally, we do not have data on biomarkers and outcomes after ICU discharge, which would have given us a more accurate depiction of the predictive capacity of these markers. Despite these limitations, we were able to investigate a wide range of inflammatory biomarkers in a population of ARDS patients with a uniform etiology.

Our findings demonstrate that certain biomarkers, particularly NLR, can predict all-cause mortality in COVID-19 ARDS patients after commencing HDS treatment. Even at later timepoints, elevated biomarker levels indicate increased mortality. To determine whether this is caused by suboptimal corticosteroid therapy or other causes, such as superinfections, additional information on co-infections and cause of death is crucial. Since patients receiving HDS are likely to have different characteristics and disease severity, which in turn influences their risk of death compared to those not receiving HDS, our analysis specifically focused on the population receiving HDS. Future studies will be required to validate these findings in other populations (ie those without HDS). Furthermore, using inflammatory biomarkers as predictors of severe outcomes may be extended to other COVID-19 treatment modalities that target the immune response, such as IL-6 inhibitors or low-dose corticosteroids. In anticipation of future infectious disease outbreaks leading to ARDS, whether the same biomarkers can predict mortality during HDS in populations with ARDS from other etiologies needs to be examined.

## Conclusion

In COVID-19 ARDS patients treated with HDS, there are significant changes in biomarker levels following the commencing HDS treatment, with an initial decrease in median levels for CRP, D-dimer, ferritin, leukocyte count, IL-6, LDH, and procalcitonin among both survivors and non-survivors. At baseline, ferritin, leukocyte count, and NLR demonstrated moderately high predictive capacity for mortality. Following the commencing HDS treatment, NLR, CRP, ferritin, IL-6, and LDH exhibited predictive capacity for mortality, particularly on days six and seven. NLR emerged as the most consistent predictor of mortality across all time points except day 1, demonstrating higher sensitivity and specificity compared to other biomarkers.

## Data Availability

The datasets used and analyzed during the current study are available from the corresponding author upon reasonable request.

## References

[1] Bellani G, Laffey JG, Pham T. Epidemiology, Patterns of Care, and Mortality for Patients With Acute Respiratory Distress Syndrome in Intensive Care Units in 50 Countries. *JAMA*. 2016;315(8):788–800. doi:10.1001/jama.2016.0291 26903337

[2] Parhar KKS, Zjadewicz K, Soo A. Epidemiology, Mechanical Power, and 3-Year Outcomes in Acute Respiratory Distress Syndrome Patients Using Standardized Screening. An Observational Cohort Study. *Ann Am Thorac Soc*. 2019;16(10):1263–1272. doi:10.1513/AnnalsATS.201812-910OC 31247145 PMC6812172

[3] Kasotakis G, Stanfield B, Haines K, et al. Acute Respiratory Distress Syndrome (ARDS) after trauma: improving incidence, but increasing mortality. *J Crit Care*. 2021;64:213–218. doi:10.1016/j.jcrc.2021.05.003 34022661

[4] WHO. Coronavirus (COVID-19) Dashboard. Accessed 6, May 2024. Available from: https://www.who.int/emergencies/diseases/novel-coronavirus-2019.

[5] Blondonnet R, Constantin JM, Sapin V, Jabaudon M. A Pathophysiologic Approach to Biomarkers in Acute Respiratory Distress Syndrome. *Dis Markers*. 2016;2016:3501373. doi:10.1155/2016/3501373 26980924 PMC4766331

[6] Castro CY. ARDS and diffuse alveolar damage: a pathologist’s perspective. *Semin Thorac Cardiovasc Surg*. 2006;18(1):13–19. doi:10.1053/j.semtcvs.2006.02.001 16766248

[7] Calfee CS, Delucchi K, Parsons PE, et al. Subphenotypes in acute respiratory distress syndrome: latent class analysis of data from two randomised controlled trials. *Lancet Respir Med*. 2014;2(8):611–620. doi:10.1016/S2213-2600(14)70097-9 24853585 PMC4154544

[8] Mehta P, Samanta RJ, Wick K. Elevated ferritin, mediated by IL-18 is associated with systemic inflammation and mortality in acute respiratory distress syndrome (ARDS). *Thorax*. 2024;79(3):227–235. doi:10.1136/thorax-2023-220292 38148147 PMC12278832

[9] Battaglini D, Lopes-Pacheco M, Castro-Faria-Neto HC, Pelosi P, Rocco PRM. Laboratory Biomarkers for Diagnosis and Prognosis in COVID-19. *Front Immunol*. 2022;13:857573. doi:10.3389/fimmu.2022.857573 35572561 PMC9091347

[10] Malik P, Patel U, Mehta D, et al. Biomarkers and outcomes of COVID-19 hospitalisations: systematic review and meta-analysis. *BMJ Evid Based Med*. 2021;26(3):107–108. doi:10.1136/bmjebm-2020-111536 PMC7493072 32934000

[11] Sinha P, Neyton L, Sarma A. Molecular Phenotypes of Acute Respiratory Distress Syndrome in the ROSE Trial Have Differential Outcomes and Gene Expression Patterns That Differ at Baseline and Longitudinally over Time. *Am J Respir Crit Care Med*. 2024;209(7):816–828. doi:10.1164/rccm.202308-1490OC 38345571 PMC10995566

[12] Figliozzi S, Masci PG, Ahmadi N, et al. Predictors of adverse prognosis in COVID-19: a systematic review and meta-analysis. *Eur J Clin Invest*. 2020;50(10):e13362. doi:10.1111/eci.13362 32726868

[13] Klok FA, Kruip M, van der Meer NJM, et al. Incidence of thrombotic complications in critically ill ICU patients with COVID-19. *Thromb Res*. 2020;191:145–147. doi:10.1016/j.thromres.2020.04.013 32291094 PMC7146714

[14] Zhou F, Yu T, Du R. Clinical course and risk factors for mortality of adult inpatients with COVID-19 in Wuhan, China: a retrospective cohort study. *Lancet*. 2020;395(10229):1054–1062. doi:10.1016/S0140-6736(20)30566-3 32171076 PMC7270627

[15] Alqahtani JS, Gonçalves Mendes R, Aldhahir A. Global Current Practices of Ventilatory Support Management in COVID-19 Patients: an International Survey. *J Multidiscip Healthc*. 2020;13:1635–1648. doi:10.2147/JMDH.S279031 33239884 PMC7680685

[16] Meduri GU, Golden E, Freire AX, et al. Methylprednisolone infusion in early severe ARDS: results of a randomized controlled trial. *Chest*. 2007;131(4):954–963. doi:10.1378/chest.06-2100 17426195

[17] Meduri GU, Schwingshackl A, Hermans G. Prolonged Glucocorticoid Treatment in ARDS: impact on Intensive Care Unit-Acquired Weakness. *Front Pediatr*. 2016;4:69. doi:10.3389/fped.2016.00069 27532030 PMC4969316

[18] Agarwal A, Hunt B, Stegemann M. A living WHO guideline on drugs for covid-19. *BMJ*. 2020;370:m3379. doi:10.1136/bmj.m3379 32887691

[19] RECOVERY Collaborative Group. Dexamethasone in Hospitalized Patients with Covid-19. *N Engl J Med*. 2021;384(8):693–704. doi:10.1056/NEJMoa2021436 32678530 PMC7383595

[20] Granholm A, Munch MW, Myatra SN. Dexamethasone 12 mg versus 6 mg for patients with COVID-19 and severe hypoxaemia: a pre-planned, secondary Bayesian analysis of the COVID STEROID 2 trial. *Intensive Care Med*. 2022;48(1):45–55. doi:10.1007/s00134-021-06573-1 34757439 PMC8579417

[21] Pinzon MA, Ortiz S, Holguin H, et al. Dexamethasone vs methylprednisolone high dose for Covid-19 pneumonia. *PLoS One*. 2021;16(5):e0252057. doi:10.1371/journal.pone.0252057 34033648 PMC8148307

[22] Russell L, Uhre KR, Lindgaard ALS. Effect of 12 mg vs 6 mg of Dexamethasone on the Number of Days Alive Without Life Support in Adults With COVID-19 and Severe Hypoxemia: the COVID STEROID 2 Randomized Trial. *JAMA*. 2021;326(18):1807–1817. doi:10.1001/jama.2021.18295 34673895 PMC8532039

[23] Tan RSJ, Ng KT, Xin CE, Atan R, Yunos NM, Hasan MS. High-Dose versus Low-Dose Corticosteroids in COVID-19 Patients: a Systematic Review and Meta-analysis. *J Cardiothorac Vasc Anesth*. 2022;36(9):3576–3586. doi:10.1053/j.jvca.2022.05.011 35715291 PMC9101704

[24] Annane D, Pastores SM, Rochwerg B, et al. Guidelines for the diagnosis and management of critical illness-related corticosteroid insufficiency (CIRCI) in critically ill patients (Part I): society of Critical Care Medicine (SCCM) and European Society of Intensive Care Medicine (ESICM) 2017. *Intensive Care Medicine*. 2017;43(12):1751–1763. doi:10.1007/s00134-017-4919-5 28940011

[25] Siddiqi HK, Mehra MR. COVID-19 illness in native and immunosuppressed states: a clinical-therapeutic staging proposal. *J Heart Lung Transplant*. 2020;39(5):405–407. doi:10.1016/j.healun.2020.03.012 32362390 PMC7118652

[26] Roche Diagnostics. Accessed 29, Jan 2024. Available from: https://diagnostics.roche.com/us/en/products/instruments/cobas-c-502-ins-2113.html.

[27] Roche Diagnostics. Accessed 29, Jan 2024. Available from: https://diagnostics.roche.com/us/en/products/instruments/cobas-e-801-ins-2202.html.

[28] Rothman KJ. No adjustments are needed for multiple comparisons. *Epidemiology*. 1990;1(1):43–46. doi:10.1097/00001648-199001000-00010 2081237

[29] Meduri GU, Headley S, Tolley E, Shelby M, Stentz F, Postlethwaite A. Plasma and BAL cytokine response to corticosteroid rescue treatment in late ARDS. *Chest*. 1995;108(5):1315–1325. doi:10.1378/chest.108.5.1315 7587435

[30] Chen X, Tang J, Shuai W, Meng J, Feng J, Han Z. Macrophage polarization and its role in the pathogenesis of acute lung injury/acute respiratory distress syndrome. *Inflamm Res*. 2020;69(9):883–895. doi:10.1007/s00011-020-01378-2 32647933 PMC7347666

[31] Calfee CS, Delucchi KL, Sinha P, et al. Irish Critical Care Trials G: **acute respiratory distress syndrome subphenotypes and differential response to simvastatin: secondary analysis of a randomised controlled trial**. *Lancet Respir Med*. 2018;6(9):691–698. doi:10.1016/S2213-2600(18)30177-2 30078618 PMC6201750

[32] Sinha P, Furfaro D, Cummings MJ. Latent Class Analysis Reveals COVID-19-related Acute Respiratory Distress Syndrome Subgroups with Differential Responses to Corticosteroids. *Am J Respir Crit Care Med*. 2021;204(11):1274–1285. doi:10.1164/rccm.202105-1302OC 34543591 PMC8786071

[33] Steinberg KP, Hudson LD, Goodman RB, et al. Blood Institute Acute Respiratory Distress Syndrome Clinical Trials N: **efficacy and safety of corticosteroids for persistent acute respiratory distress syndrome**. *N Engl J Med*. 2006;354(16):1671–1684. 16625008 10.1056/NEJMoa051693

[34] Peukert K, Sauer A, Seeliger B. Increased Alveolar Epithelial Damage Markers and Inflammasome-Regulated Cytokines Are Associated with Pulmonary Superinfection in ARDS. *J Clin Med*. 2023;12(11):3649. 37297845 10.3390/jcm12113649 PMC10253810

[35] Chen Z, Zhan Q, Huang L, Wang C. Coinfection and superinfection in ICU critically ill patients with severe COVID-19 pneumonia and influenza pneumonia: are the pictures different? *Front Public Health*. 2023;11:1195048. doi:10.3389/fpubh.2023.1195048 37711242 PMC10497876

[36] Wagner KKL, Corda D, Steinmayr A, et al. CRP/Neopterin Ratio and Neuropsychiatric Symptoms in Patients with Different Forms of Pneumonia: results of a Pilot Study. *Microorganisms*. 2024;12(6):1099. 38930481 10.3390/microorganisms12061099 PMC11205953

[37] XS W, Jiang D, Gao L, et al. Clinical characteristics and predictive value of lower CD4(+)T cell level in patients with moderate and severe COVID-19: a multicenter retrospective study. *BMC Infect Dis*. 2021;21(1):57. doi:10.1186/s12879-020-05741-w 33435865 PMC7803000

[38] Seyfi S, Azadmehr A, Ezoji K, et al. Mortality in ICU COVID-19 Patients Is Associated with Neutrophil-to-Lymphocyte Ratio (NLR): utility of NLR as a Promising Immunohematological Marker. *Interdiscip Perspect Infect Dis*. 2023;2023:9048749. doi:10.1155/2023/9048749 38025794 PMC10653951

[39] Li X, Liu C, Mao Z, et al. Predictive values of neutrophil-to-lymphocyte ratio on disease severity and mortality in COVID-19 patients: a systematic review and meta-analysis. *Crit Care*. 2020;24(1):647. doi:10.1186/s13054-020-03374-8 33198786 PMC7667659

[40] McGregor BA, Murphy KM, Albano DL, Ceballos RM. Stress, cortisol, and B lymphocytes: a novel approach to understanding academic stress and immune function. *Stress*. 2016;19(2):185–191. doi:10.3109/10253890.2015.1127913 26644211 PMC4837014

[41] Cacciapuoti S, De RA, Gelzo M, et al. Immunocytometric analysis of COVID patients: a contribution to personalized therapy? *Life Sci*. 2020;261:118355. doi:10.1016/j.lfs.2020.118355 32871183 PMC7456265

[42] Saffar AS, Ashdown H, Gounni AS. The molecular mechanisms of glucocorticoids-mediated neutrophil survival. *Curr Drug Targets*. 2011;12(4):556–562. doi:10.2174/138945011794751555 21504070 PMC3267167

[43] Tornatore KM, Venuto RC, Logue G, Davis PJ. CD4+ and CD8+ lymphocyte and cortisol response patterns in elderly and young males after methylprednisolone exposure. *J Med*. 1998;29(3–4):159–183. 9865456

